# Multi-Scenario Validation and Assessment of a Particulate Matter Sensor Monitor Optimized by Machine Learning Methods

**DOI:** 10.3390/s24113448

**Published:** 2024-05-27

**Authors:** Hao Tang, Yunfei Cai, Song Gao, Jin Sun, Zhukai Ning, Zhenghao Yu, Jun Pan, Zhuohui Zhao

**Affiliations:** 1 NHC Key Laboratory of Health Technology Assessment, Key Laboratory of Public Health Safety of the Ministry of Education, Department of Environmental Health, School of Public Health, Fudan University, Shanghai 200032, China; 22111020056@m.fudan.edu.cn (H.T.);; 2 Department of General Management and Statistics, Shanghai Environment Monitoring Center, Shanghai 200235, China; yfcai@sthj.shanghai.gov.cn (Y.C.);; 3 School of Environmental and Chemical Engineering, Shanghai University, Shanghai 200444, China; njulegao@163.com (S.G.);; 4 Shanghai Key Laboratory of Meteorology and Health, Typhoon Institute/CMA, IRDR International Center of Excellence on Risk Interconnectivity and Governance on Weather/Climate Extremes Impact and Public Health WMO/IGAC MAP-AQ Asian Office Shanghai, Fudan University, Shanghai 200438, China

**Keywords:** air pollution, exposure assessment, environmental monitoring, light-scattering method

## Abstract

Objective: The aim was to evaluate and optimize the performance of sensor monitors in measuring PM_2.5_ and PM_10_ under typical emission scenarios both indoors and outdoors. Method: Parallel measurements and comparisons of PM_2.5_ and PM_10_ were carried out between sensor monitors and standard instruments in typical indoor (2 months) and outdoor environments (1 year) in Shanghai, respectively. The optimized validation model was determined by comparing six machining learning models, adjusting for meteorological and related factors. The intra- and inter-device variation, measurement accuracy, and stability of sensor monitors were calculated and compared before and after validation. Results: Indoor particles were measured in a range of 0.8–370.7 μg/m^3^ and 1.9–465.2 μg/m^3^ for PM_2.5_ and PM_10_, respectively, while the outdoor ones were in the ranges of 1.0–211.0 μg/m^3^ and 0.0–493.0 μg/m^3^, correspondingly. Compared to machine learning models including multivariate linear model (ML), K-nearest neighbor model (KNN), support vector machine model (SVM), decision tree model (DT), and neural network model (MLP), the random forest (RF) model showed the best validation after adjusting for temperature, relative humidity (RH), PM_2.5_/PM_10_ ratios, and measurement time lengths (months) for both PM_2.5_ and PM_10_, in indoor (R^2^: 0.97 and 0.91, root-mean-square error (RMSE) of 1.91 μg/m^3^ and 4.56 μg/m^3^, respectively) and outdoor environments (R^2^: 0.90 and 0.80, RMSE of 5.61 μg/m^3^ and 17.54 μg/m^3^, respectively), respectively. Conclusions: Sensor monitors could provide reliable measurements of PM_2.5_ and PM_10_ with high accuracy and acceptable inter and intra-device consistency under typical indoor and outdoor scenarios after validation by RF model. Adjusting for both climate factors and the ratio of PM_2.5_/PM_10_ could improve the validation performance.

## 1. Introduction

A study on the global burden of disease showed that the largest increase in health burden was due to environmental particulate pollution from 2010 to 2019 [1]. In China, for example, the number of premature deaths by fine particulate matter (PM_2.5_) was 1.27 million, and that of chronic obstructive pulmonary disease in adults was 119,167, lung cancer 83,976, ischemic heart disease 39,266, and stroke 679,906 [2]. People spend around 80–90% of their lifetime in an indoor environment [3,4,5]. Indoor PM_2.5_ has been recently listed as the top risk factor contributing the largest disease burden compared to any other air pollutant in China [6,7] and in other countries [8,9]. However, assessment on exposure to particles in the population has often been underestimated due to lack of large-scale measurements in the indoor environment [10]. An accurate assessment of human exposure to airborne particles in both outdoor and indoor environments is needed, especially PM_2.5_ and inhalable particulate matter (PM_10_), since they can penetrate the bronchial and trachea regions and deposit at lung bifurcations and even deep into lung the alveoli [11,12]. With a decrease in outdoor airborne particulate matter (PM) in China and in other areas in the world [13,14], the measurement of indoor particles, possibly multiples times higher than those in outdoor environments [15,16,17,18], is becoming more prominent and warrants improved strategy in PM exposure assessment. 

At present, the measurement of outdoor particulate matter is mainly by gravimetric method, micro-oscillation balance method, and β-ray absorption method, while in indoor environments, sensor-based real-time measurements by light-scattering methods have developed rapidly. With fast response, high sensitivity, small sizes, and low costs [19], sensor monitors for particles have great potential in building up the real-time large-scale evaluation network of exposure to particles, integrating both indoor and outdoor environments [20,21,22]. However, many monitors are dominant in commercial use and lack scientific validation of their accuracy, reliability, and durability in both indoor and outdoor scenarios. 

The performance of sensor monitors, evaluated by intra-consistency and inter-consistency in comparison with reference instruments, is key to measurement data quality, as they are likely to under- or overestimate the true particle levels, which further produces bias in assessments of human exposure levels. At present, there are certain validation studies on PM_2.5_ sensor monitors and their influencing factors [23,24,25], but few were completed in rich scenarios with different emissions sources both in outdoor and indoor environments. Previous studies have shown that sensor performance is affected by relative humidity (RH) and temperature, but fewer studies have actually corrected for and applied this finding to the measurement’s validation [11,12]. In our pilot tests, we found that particle size distribution, e.g., the ratio of PM_2.5_ to PM_10_, especially in the outdoor environment, largely influenced the measurements of the mass concentrations in sensor monitors, which needs to be considered in validation. Additionally, a large amount of data will be generated in real-time sensor monitors in seconds or in minutes. Advanced regression methods such as machine learning models are expected due to their better capacity for handling large amounts of data [26,27]. Even within the same field as machine learning models, there is a great variety in the strengths and calculation capacities of the methods, which warrants further comparisons [28,29,30,31]. Integrating all these factors into the validation of sensor monitors will contribute to promoting the performance of sensor-based particle measurements. 

In this study, a new sensor-based real-time particulate monitor was tested and validated on its measurements of PM_2.5_ and PM_10_ against standard instruments. The tests were completed in seven scenarios, with six in an indoor environment (one in offices for 2 months with no obvious indoor emission source and five in residences with typical indoor emission sources of cooking, smoking, and burning) and one in an outdoor environment (for 12 continuous months). Six machine learning models were explored on their performance in validating the measurements covering the above indoor and outdoor scenarios. The influencing environmental factors, such as environmental temperature, RH, PM_2.5_/PM_10_ ratios, and measurement time lengths, were investigated. This study is of significance in promoting the application of low-cost sensor monitors of particles in scientific research with reliable data, which further promotes the affordable and accurate assessment of human exposure to particles, especially in large-scale population-based environmental epidemiological studies.

## 2. Materials and Methods

### 2.1. Particulate Matter Instruments

#### 2.1.1. Sensor Monitors

The sensor-based monitor (FS-Air 2.0, Fudan University, Shanghai, China) is equipped with PMSA003 particle sensors (Plantower Company, Shenzhen, China), a temperature sensor (SHT30, Sensirion Company, Stäfa, Switzerland), and an RH sensor (SHT30, Sensirion Company, Switzerland). The size of the particulate sensor is 38 × 35 × 12 mm^3^. It utilizes a fan to draw air into a cavity, where it is exposed to a laser-induced light, and the 90° scattered light is detected by a photodiode detector [32,33,34]. The PMSA003 sensor is a universal digital particle sensor based on the principle of laser scattering, which can continuously collect and calculate the number of suspended particles in different particle sizes per unit volume (the particle concentration distribution) and then convert it into mass concentration (mass concentration (μg/m^3^) = number concentration (particles/cm^3^) × particle mass density (μg/cm^3^) × air density (g/m^3^)). In this study, both PM_2.5_ and PM_10_ were measured and validated.

#### 2.1.2. Reference Instruments

In the outdoor environment, the tapered element oscillating microbalance (TEOM) was used to validate the sensor measurements of PM_2.5_ and PM_10_. In the indoor environment, a light-scattering particle monitor, the Environmental DustTrak online particulate matter monitor (EDTPM_2.5_-CN/EDTPM10-CN/EDTDRX-CN, TSI, Dallas, TX, USA, abbreviated at “TSI instrument”), was applied as a reference instrument for PM_2.5_ and PM_10_. Its measurements as reference were pre-validated against the gravity methods by TEOM for PM_10_ and by MiniVol Tactical Air Sampler for PM_2.5_, respectively. 

The TEOM (Thermos Scientific™ 1405 TEOM™, Waltham, MA, USA) is an online “gravimetric” particulate matter monitoring instrument that uses tapered element micro-oscillating balance technology. The pumped air went through the filters at a constant flow rate of 3 L/min, and the aimed particulate matter was captured on the filters. The filters were continuously weighed, and the hourly mass concentrations of PM_2.5_ and PM_10_ were recorded. 

The Environmental DustTrak online particulate matter monitor (EDTPM_2.5_-CN/EDTPM10-CN/EDTDRX-CN, TSI) served as a reference instrument for indoor PM_2.5_ and PM_10_ measurements. It is based on the DustTrak technology design using a 90° light-scattering laser photometer. The sampling flow was 3 L/min, and both PM_2.5_ and PM_10_ were measured and recorded per minute. TSI instrument was pre-validated to TEOM on PM_10_ and to MiniVol gravity method on PM_2.5_ by around 2-month continuous parallel sampling, respectively. In the validation against TEOM, a total of 1202 h (around 50 days) of parallel continuous measurements were obtained, while for validation against the MiniVol gravity method, a total of 49 daily filter samples of PM_2.5_ were collected and weighted. Linear regression analysis was performed to test the measurements of PM_2.5_ and PM_10_ by TSI as reference for sensor monitors. 

The MiniVol Tactical Air Sampler is a low-volume, battery-powered, filter-based PM sampler. The basic principle is to extract a volume of air at a constant speed and pass it through a cyclone to collect the PM_2.5_ particles. Size-sorted particles are captured on filters (37 mm Teflon) that are weighed at constant temperature and RH to determine the mass of PM_2.5_ in the air pumped through the sampler (“gravimetric method”). Each sample by the MiniVol was collected after a 24 h continuous sampling at a flow rate of 3 L per minute (3 L/min). 

### 2.2. Evaluation of Sensor Monitors in Outdoor and Indoor Environments

#### 2.2.1. Evaluation of Sensor Monitors in Outdoor Environment

Parallel measurements of real-time PM_2.5_ and PM_10_ were performed in the outdoor environment between five randomly selected sensor monitors (FS-Air 2.0) and a TEOM instrument from February 2021 to February 2022 for 12 months continuously. All instruments were installed in a traffic air pollution monitoring station in Caoxi Road, Xuhui District, Shanghai. Shanghai is located in the middle and east of China, with a typical monsoon climate. There are four distinct seasons in Shanghai. In summer, the temperature normally varies between 30 °C and 35 °C or even above. In winter, the temperature is usually around above 0 °C and varies between 1 and 10 °C but sometimes records degrees in the negative (http://www.weatheronline.co.uk/, accessed on 10 April 2024).

Hourly averages were calculated and compared between sensor monitors and TEOM. This comparison was made in a whole-year dataset and in two subgroups of days with PM_2.5_ or PM_10_ as the primary pollutants, respectively. The grouping into days with either PM_2.5_ or PM_10_ as the primary pollutant was determined by comparing the individual air quality index (IAQI) according to the calculation method by the Ambient Air Quality Standard in China [35]. In our study, days with higher IAQI of PM_2.5_ than that of PM_10_ were labelled as days with PM_2.5_ as the primary pollutant days and vice versa [36]. 

#### 2.2.2. Evaluation of Sensor Monitors in Indoor Environment

The indoor measurements were conducted in an office and a residential building in Xuhui District, Shanghai, China. The office had no obvious emission source of particles, and the residential building was tested under five scenarios of typical emission sources. The indoor measurements were performed from May to August in 2021 in Shanghai, China. 

In the office and residence, parallel measurements were performed between five randomly selected sensor monitors (FS-Air 2.0) and the TSI instrument. All instruments were placed in the same point in the center of the room and at the same height, about 1.5 m high above the ground, which ensured that they were the same distance away from the pollution source. In the office, measurements were taken for 2 continuous months, and the hourly averages were calculated and compared. In the residence, five emission scenarios were tested, including (1) cooking, (2) smoking, (3) burning of mosquito-repellent incense, (4) burning of worship incense, and (5) burning of candles. Each scenario took around 2–2.5 h, during which doors and windows were closed to avoid wind interferences. Between any two scenarios, the doors and windows were reopened to fully ventilate the room until the indoor particle levels decreased to background levels.

### 2.3. Statistical Analysis

#### 2.3.1. Intra-Consistency within Sensor Monitors

The internal consistency between sensors was evaluated by intra-device correlation coefficient (r, Pearson correlation coefficient) and intra-device variability (IDV). The IDV was the average standard deviation (*V*) across parallel sensor monitors, which was calculated as follows:

$$V_{j}=\sqrt{\frac{{\sum_{i=1}^{n}\left(C_{ij}-\bar{C_{j}}\right)}^{2}}{2}}$$

$$V=\frac{1}{m}\times \sum_{j=1}^{m}V_{j}$$

*V_j_* is the mean standard deviation of each sensor monitor; *V* is the average standard deviation across all parallel sensor monitors; $C_{ij}$ is the value of device *i* at *j* measurement; $\bar{C_{j}}$ is the mean value of instruments; *m* is the number of devices; *n* is the number of times.

#### 2.3.2. Inter-Consistency between Sensor Monitors and Reference Instruments

We used the coefficient of determination (*R*^2^) and root-mean-square error (*RMSE*) to evaluate the consistency between sensor monitors and the reference instruments and to evaluate the fitting effect of the model.

*R*^2^ was calculated, and the sample size was corrected as follows:

$$R^{2}=1-\left(1-\frac{\sum {\left({\hat{y}}_{i}-\bar{y}\right)}^{2}}{\sum {\left(y_{i}-\bar{y}\right)}^{2}}\right)*(n-1)/(n-p-1)$$

The ${\hat{y}}_{i}$ is the y estimate into which we put the square factor. $\bar{y\;}$ is the average of the actual *y* values. *p* is the number of variables, and *n* is the number of samples.

*RMSE* was calculated as follows:

$$RMSE=\sqrt{\frac{1}{m}\sum_{i=1}^{m}{\left(y_{i}-{\hat{y}}_{i}\right)}^{2}}$$

where *m* is the effective sample size. The ${\hat{y}}_{i}$ is the *y* estimate into which we put the square factor. 

The accuracy of sensor measurements was evaluated by mean relative error (*MRE*, also called mean absolute percentage error (MAPE)), and the calculation of *MRE* is as follows:

$$MRE=\frac{1}{n}\sum_{i=1}^{n}\frac{\left|C_{t,i^{-C}r,i}\right|}{C_{r,i}}\times 100\%$$

where *n* is the effective sample size; *C_t,i_* is the mass concentration measured by the sensor; *C_r,i_* is the mass concentration measured by the reference instrument.

#### 2.3.3. Validation and Optimization of Sensor Monitors by Machine Learning Methods

The validation of PM_2.5_ and PM_10_ measurements by sensor monitors (FS-Air 2.0) was performed and optimized by machining learning methods against reference measurements in indoor and outdoor environments, respectively. The regression R^2^ was compared among six machine learning methods using ten-fold cross-validation in the univariate model. Ten-fold cross-validation is commonly used to evaluate the performance of machine learning models. It divides the dataset into 10 equal parts or “folds”. The process is then repeated 10 times, with each fold being used as the test set once, while the remaining nine folds are used as the training set. The six machine learning models were the multiple linear model (ML), K-nearest neighbor model (KNN), support vector machines model (SVM), decision tree model (DT), random forest model (RF), and neural network model (multi-layer perceptron, MLP). ML was the most simple and basic model, which was used for comparison with other more advanced methods. KNN is a non-parametric and lazy learning algorithm that stores the training dataset and uses distance functions to predict the category of a new data point. The algorithm calculates the distance between the new data point and all the points in the training dataset. It then identifies the “K” nearest neighbors and assigns the most common class among these neighbors to the new point. SVM works by mapping the input features into a higher-dimensional space where a linear separation is possible. When linear separation is not possible, a kernel trick is used to implicitly map the data into a higher-dimensional space without explicitly performing the mapping. DT model is a simple yet powerful model that uses a tree-like structure to represent decisions and their possible consequences. Each internal node represents a feature, each branch represents a decision rule, and each leaf node represents an outcome. The model is built by recursively splitting the data into subsets based on the feature values, aiming to maximize the information gain or minimize the Gini impurity at each step. RF is an ensemble learning method that operates by constructing a multitude of decision trees at training time and outputting the class that is the mode of the classes (classification) or mean prediction (regression) of the individual trees. Each tree in the forest is built from a random subset of the training data (bagging) and a random subset of the features (feature subset selection). RF has the advantage of reducing the risk of overfitting and improving the models’ performance by combining the results of multiple decision trees. MLP is a class of feedforward artificial neural networks that consists of at least three layers: an input layer, one hidden layer, and an output layer. Except for the output layer, each layer consists of neurons that have a non-linear activation function. MLP uses a supervised learning technique called backpropagation for training the model, which involves forwarding the input through the network and then calculating the error at the output, followed by backward propagation of this error to adjust the weights.

First, the model with the highest R^2^ was selected as the potentially optimized model in the univariate regression analysis mentioned above. Second, we set up multivariate regression models and stepwise added temperature, RH, measuring time lengths (months), and k value (ratio of PM_2.5_ to PM_10_) as covariates both in the basic ML model and the potentially optimized model. The regression R^2^ values were compared between the two models at each step of adding a new covariate. 

#### 2.3.4. Marginal Effects of Explanatory Features on the Validation Model

After determining the final validation model, a partial dependence plot (PDP), a machine learning explanatory tool, was applied to illustrate the marginal effects of explanatory features on the prediction of the model. The PDP showed whether the relationship between target effects and explanatory features was linear, monotonic, or in a more complicated style. 

Finally, the improved sensor measurement data after validation by the best model were compared to the data after conventional linear regression validation on their intra-device correlation coefficient (r), intra-device variability (IDV), R^2^, MRE%, and RMSE, respectively. In the whole analysis, PYTHON 3.8.5 software was applied for statistical analysis, where Scikit-learn libraries were used for machine learning.

## 3. Results

### 3.1. Applicability of TSI as a Reference Instrument for Sensor Monitors

The average PM_10_ by TSI (69.9 ± 38.7 μg/m^3^) was very close to that by TEOM (67.3 ± 36.8 μg/m^3^), with its R^2^ and RMSE reaching 0.91 and 5.7 μg/m^3^, respectively (Table S1). The average PM_2.5_ concentration by TSI (20.9 ± 12.0 μg/m^3^) was the same or close to that of the gravity method by MiniVol TAS instrument (19.3 ± 99.1 μg/m^3^), with the R^2^ and RMSE reaching 0.88 and 4.9 μg/m^3^, respectively (Table S2). The results showed that PM_2.5_ and PM_10_ measurements by TSI had good comparability with the gravity methods and were applicable as reference for sensor monitors.

### 3.2. Parallel Measurements between Sensor Monitors and TEOM in Outdoor Environment

**Figure 1:**
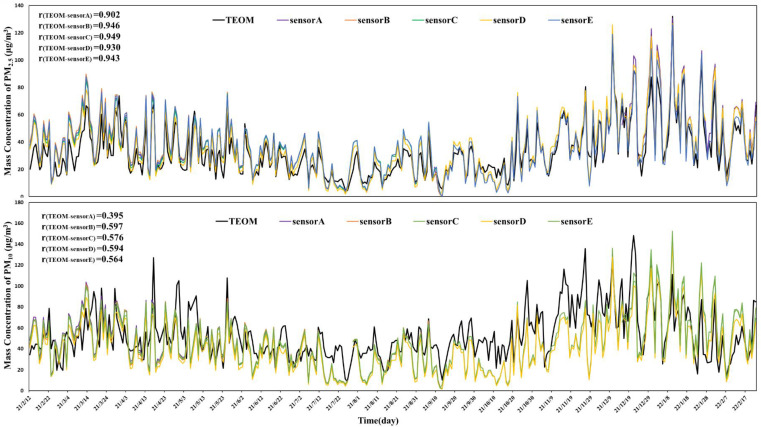
Daily averages of PM_2.5_ and PM_10_ (µg/m^3^) by five randomly selected sensors and the reference TEOM instrument (black line) at the Caoxi Road Traffic Station, Xuhui District, Shanghai, from February 2020 to February 2021.

In the year-round outdoor measurements, the daily averages of PM_2.5_ and PM_10_ by sensor monitors were in a synchronous variation pattern with the TEOM measurements (Figure 1). Between the five sensor monitors, a good correlation was observed (r ≥ 0.99). The average PM_2.5_ raw data by sensor monitors (FS-Air 2.0) were numerically higher (*p* < 0.05) than that of TEOM, and PM_10_ was lower than that of TEOM (*p* < 0.05) (Table 1). The raw measurements of PM_2.5_ and PM_10_ did not satisfy the field measurement requirements and needed to be calibrated.

According to TEOM, the annual ambient concentrations of PM_2.5_ and PM_10_ were 33.3 ± 23.6 μg/m^3^ and 54.7 ± 38.4 μg/m^3^, respectively (Table 1). The winter season had the highest levels of PM_2.5_ (46.2 ± 30.1 μg/m^3^) and PM_10_ (60.9 ± 37.8 μg/m^3^), while summer had the lowest levels (PM_2.5_: 21.1 ± 12.6 μg/m^3^ and PM_10_: 38.8 ± 18.2 μg/m^3^). The R^2^ of the one-year outdoor measurements of PM_2.5_ and PM_10_ by sensor monitors was 0.79 and 0.26, respectively, regressed against the TEOM measurements. The local climate had obvious seasonal variations, and its influences could not be neglected. The whole-year daily average temperature was 19.5 ± 8.5°C, varying in a range of 0.4~38.0°C, and the daily average RH was 67.2 ± 14.6% in a range of 23.7%~93.9%. Between the randomly selected sensors, there was a good internal consistency on temperature and humidity (r = 0.99).

The outdoor measurements were grouped into days with PM_2.5_ or PM_10_ as the primary pollutant, respectively. We found that in days with PM_2.5_ pollution as the primary pollutant, the R^2^ of the linear model for PM_2.5_ out of sensor monitors was higher than that of days when PM_10_ was the primary pollutant (0.74 vs. 0.65). For PM_10_, there was a slight increase in the R^2^ of PM_10_ by sensor monitors on days when PM_10_ pollution was the primary pollutant (0.36 vs. 0.30) (Table S3). 

### 3.3. Parallel Measurements between Sensor Monitors and TSI Instrument in Indoor Environment

In an office with no obvious indoor emission sources of particles

We compared PM_2.5_ and PM_10_ by the sensors and TSI in the office for around two months using linear models. The average PM_2.5_ (17.6 ± 13.3 μg/m^3^ vs. 19.9 ± 14.3 μg/m^3^) and PM_10_ (20.4 ± 16.0 μg/m^3^ vs. 27.1 ± 18.8 μg/m^3^) by sensor monitors were very close to those by TSI in linear models (Table 2), respectively. The R^2^ between two types of instruments was 0.66 and 0.64 for PM_2.5_ and PM_10_, respectively. The above measurements were completed at an average temperature of 31.2 ± 1.1°C, with the average RH and atmospheric pressure at 69.2 ± 4.3% and 100.3 ± 0.5 Pa, respectively. There was still good internal consistency of temperature and humidity between the five randomly selected sensors (r = 0.99). Between the five sensor monitors, PM_2.5_ and PM_10_ showed good consistency (r = 0.99) (Figure 1). 

In residence under six typical indoor emission scenarios

**Figure 2:**
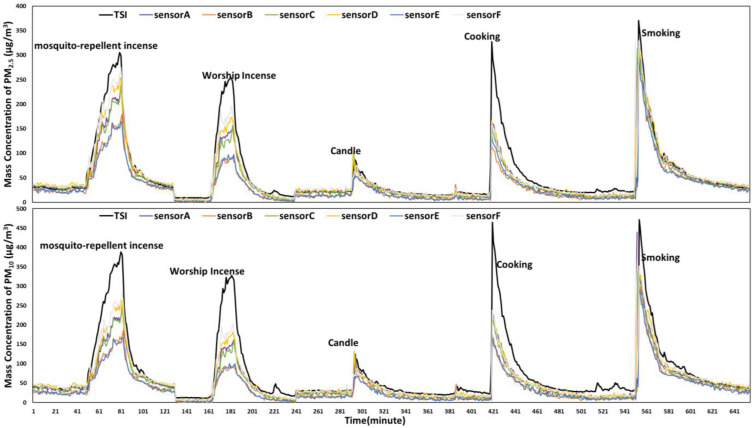
Parallel measurements of PM_2.5_ and PM_10_ (µg/m^3^) by six sensor monitors and one TSI under five scenarios in an indoor environment.

The performance of sensor monitors in the indoor environment was tested under six typical scenarios, each with a different emission source (Table 2). The results showed that the measurements of PM_2.5_ and PM_10_ by sensor monitors were highly consistent with TSI (all R^2^ ≥ 0.96). The sensor monitors showed the indoor particles were in a range of 2.7 μg/m^3^ to 308.2 μg/m^3^ for PM_2.5_ and 3.8 μg/m^3^ to 471.6 μg/m^3^ for PM_10_, respectively. The top peak concentration was observed in smoking and cooking scenarios (Figure 2), and the lowest scenario was in candle burning. 

### 3.4. Validation of Sensor Monitor Data Optimized by Machine Learning Models

The above parallel measurement data were polled to explore for the optimized validation model for sensor monitors against the reference instrument for PM_2.5_ and PM_10_ in outdoor and indoor environments, respectively (Table 3). Among all the models, the RF model adjusting for temperature, RH, month, and k value (the ratio of PM_2.5_/PM_10_) had the highest R^2^ of 0.90 and 0.80 for outdoor PM_2.5_ and PM_10_ and 0.97 and 0.91 for indoor PM_2.5_ and PM_10_, respectively. The MRE of PM_2.5_ and PM_10_ was less than 25%, and the RMSE of PM_2.5_ and PM_10_ was less than 18 μg/m^3^ (Table S5).

**Figure 3:**
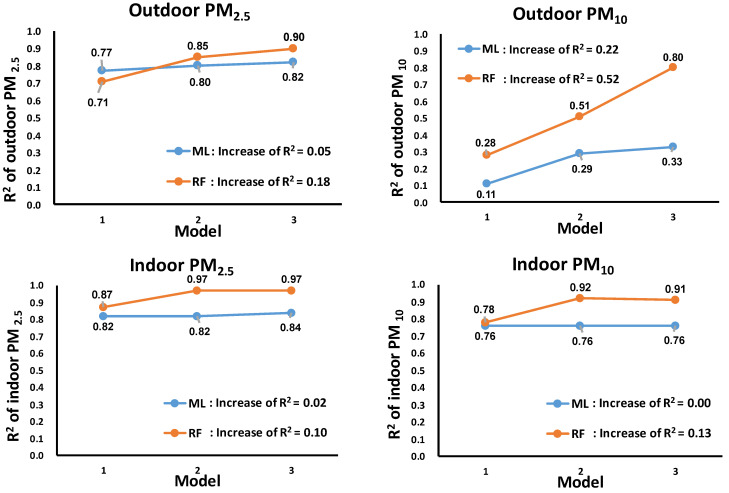
The regression R^2^ of PM_2.5_ and PM_10_ by sensor monitors against reference data, adjusting for covariates in the ML and RF models (10-fold cross-validation). ML, multiple linear model; RF, random forest; k value: PM_2.5_/PM_10_ × 100%. Model 1: yPM_ref_~XPM_sensor_; model 2: yPM_ref_~XPM_sensor_+T+RH+measurement time lengths (months); model 3: yPM_ref_~XPM_sensor_+T+RH+measurement time lengths(months)+k.

The construction and optimization of the above validation model are illustrated in Figure 3. From model 1 (univariate regression with sensor monitor data as the only independent variable) to model 2 (adjusting for temperature, RH, and measurement time length (months)), there were large improvements of model fitting both in the indoor and outdoor environments. From model 2 to model 3, with the addition of k value, there was still an obvious increase in the R^2^ in the outdoor environment, while that in the indoor environment did not show big differences. In either model 1, model 2, or model 3, the RF machine learning model always showed better performance than the ML model, which indicates that its good performance was independent of covariates. The details in each step of model construction for both indoor and outdoor environments are shown in Table S4 and the scatter plots shown in Figure S1. Generally, data at higher levels tended to be underestimated in the ML models but could be better fit in the RF models.

### 3.5. Marginal Effects of Explanatory Features on the Validation Model

**Figure 4:**
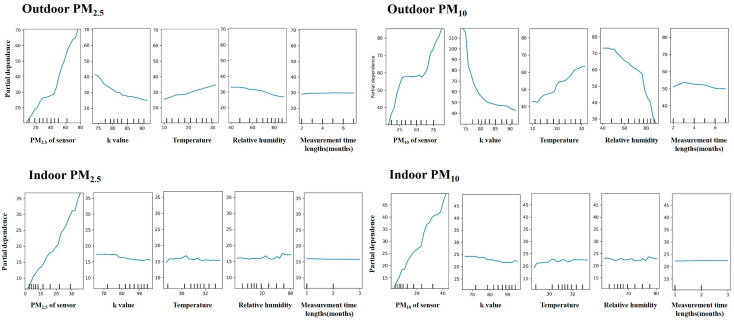
One-way partial dependence plot in the optimized RF model for PM_2.5_ and PM_10_ sensor monitor measurements, including airborne PM_2.5_, k value, temperature, RH, and measurement time lengths (months). k value: PM_2.5_/PM_10_ × 100%.

We used a one-way partial dependence plot to explore for the marginal effects of each factor in the RF model to examine its influence on the model fitting of sensor monitor measurements in outdoor and indoor environments, respectively. In Figure 4, sensor monitor data on particles had the largest quasi-linear relationship with the model prediction either for PM_2.5_ or PM_10_. The model fitting of outdoor PM_10_ was largely influenced by k value and RH in a negative way but by temperature in a positive way. This characteristic was similar for outdoor PM_2.5_ but to a much milder extent. For indoor measurements of PM_2.5_ and PM_10_, there were almost no influences by temperature, RH, or k value. In both indoor and outdoor environments, the measurement time lengths (months) did not influence nearly any model predictions, indicating the good durability of sensor monitors.

### 3.6. Evaluation of Sensor Performance after Optimal Validation

**Figure 5:**
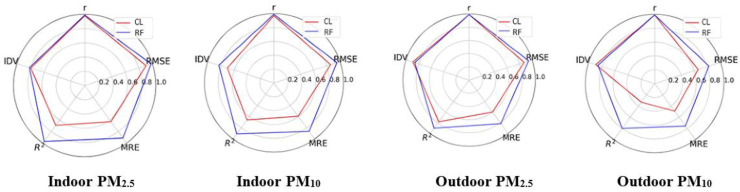
Evaluation of PM_2.5_ and PM_10_ measurements after validation by random forest model and linear regression model in both indoor and outdoor environments (10-fold cross-validation). A total of five indices were transferred to a scale of 0–1. Score 1 is the best performance and score 0 the worst. CL: validated by conventional linear regression models for all data; RF: validated by ten-fold cross-validation; r is intra-device correlation coefficient. Score 1 is the highest correlation and 0 is the lowest; IDV is intra-device variability. Score 1 means the intra-variability was 0 μg/m^3^, while for score 0, it was 10 μg/m^3^; R^2^ is the coefficient of determination. Score 1 is the highest level and score 0 the lowest; MRE, mean relative error. Score 1 is 0%, and score 0 refers to 100%; RMSE, root-mean-square error. Score 1 is 0%, and score 0 is 100%.

By comparing the sensor measurement performance before and after RF validation, we evaluated the improvement of sensor monitor measurements (Figure 5, Table S5). Generally, the measurements of PM_2.5_ and PM_10_ were largely improved after RF validation. Compared to PM_10_, the measurements of PM_2.5_ were more precise and accurate both in indoor and outdoor environments. Compared to the outdoor environment, the measurements were more stable and robust in the indoor environment.

In detail, after validation, the intra-device correlation coefficient (r = 0.99) was close to 1, and the intra-device variability (IDV), the indicator of consistency between the sensors, was ≤2 μg/m^3^ for PM_2.5_ or PM_10_ both in indoor and outdoor scenarios. The R^2^ of PM_2.5_ and PM_10_ sensor monitors in the outdoor environment reached 0.90 (15% increase) and 0.80 (142% increase), respectively, while in the indoor environment, they were 0.97(41% increase) and 0.91(38% increase), respectively. The MRE of PM_2.5_ and PM_10_ sensor monitor measurements were lower than 25% and 15% in the outdoor and indoor environments, respectively. The RMSE of two size fractions of particles, both indoors and outdoors, was close to or lower than 5 μg/m^3^, except for PM_10_ in the outdoor environment (17.54 μg/m^3^).

## 4. Discussion

In this study, parallel measurements of PM_2.5_ and PM_10_ by sensor monitors (FS-Air 2.0) and reference instruments were conducted for one year in an outdoor environment and for 2 months in an indoor environment under a series of typical emission scenarios. After an optimized validation by machining learning methods, the sensor monitors reached a high precision and accuracy for PM_2.5_ (R^2^ > 0.9, MRE < 20%, RMSE around ≤5 μg/m^3^) and PM_10_ measurements (R^2^ > 0.8, MRE < 25%, RMSE ≤ 18 μg/m^3^), respectively. Compared to outdoor measurements, the indoor measurements of PM_2.5_ and PM_10_ were more precise and stable. Our findings can help promote the field applications of sensor monitors in micro-environment measurements and in individual exposure assessment for improved environmental epidemiology research.

Light-scattering sensor monitors have been widely applied in field measurements due to their advantages of low cost, small size, portability, and noiselessness [37,38]. However, their measurements in accuracy, stability, and durability are challenges in its application in scientific research. Finding an optimized validation method is important to ensure these monitors’ measurement performance. The coefficient of determination (R^2^) is a key parameter to evaluate whether a sensor has a robust response to particulate matter. In this study, compared with the conventional multi-linear (ML) model [23,26,39], the RF model had the best performance with the highest R^2^ when adjusting for the same influencing factors. The better performance of the RF model in validation was consistent with previous studies’ findings that the R^2^ of the random forest model (R^2^ = 0.81 [24], 0.85 [38], and 0.98 [40]) was higher than that of the multivariate linear model (R^2^ = 0.50 [24], 0.83 [38], and 0.87 [40]), respectively.

In this study, the optimal RF model was screened out after multiple comparisons with other machine learning models, such as support vector machines (SVM), neural networks (multi-layer perceptron, MLP), K-nearest neighbor (KNN), and decision tree (DT). As far as we know, there has been no comparison within multiple machine learning models [24,38,40,41], although there were studies using RF models or neural network models. The best fitting by the RF model might be due to the following points. First, the RF model can handle the complex non-linear relationships between PM_2.5_ and PM_10_ measurement values and meteorological factors. It has a strong ability to deal with non-linear problems. Second, it has strong generalization capacity by integrating multiple decision trees, reducing the risk of overfitting and adapting to new data. Third, the RF model can capture the interactions between features, e.g., between particles and meteorological factors, which are crucial for improving predictive accuracy. For other machine learning models, SVM performed the next best after the RF model in the PM_2.5_ fitting in the outdoor scene (Table 3). SVM is especially suitable for handling data in high-dimensional spaces because it searches for the optimal hyperplane in the feature space to separate the data. It also shows good performance in small sample cases because it relies on finding key support vectors instead of the entire data set. DT performed close to the RF model in the PM_2.5_ and PM_10_ fitting in the indoor environment (Table 3). DT is easy to understand, as it can visualize the decision-making process, which is suitable for scenarios that require rule extraction. Compared with DT regression (weak machine learning method), RF regression is an integrated machine learning method and is able to set up multiple weak machine learning methods to present better-fitting results. In addition, the KNN model was not the optimal one in our study, but it also showed a good fitting of sensor monitor data compared to the traditional methods. Compared with the MLP model, the SVM and MLP models showed no significant improvement in performance.

The optimal validation needs to fully consider the influencing factors [42,43]. We adjusted temperature, RH, ratio of PM_2.5_/PM_10_ (k value), and measurement time length (in months) in the validation model. Outdoor measurements had more complex conditions, such as more extreme fluctuations of temperature, larger variations of RH and precipitation, and more complicated composition of emission sources of pollutants than the indoor scenario. This makes it more difficult for sensors to maintain high accuracy and stability at different pollution levels. Comparatively, the measurements of PM_2.5_ and PM_10_ in the indoor environment were less influenced by temperature, RH, and the k value, while measurements in the outdoor environment were largely influenced by temperature, RH, and the k value. These differences were obvious in the one-way partial dependence analysis (Figure 4). It indicated that the particle measurements by sensor monitors in the outdoor environment could largely vary in different seasons under different climates with varied temperatures and RH levels if not properly validated in advance. To enhance the reliability of outdoor sensors, we proposed to increase the validation data for model training; maintain regular calibration, especially in the outdoor measurement, to adapt to the ever-changing environmental conditions; and even provide rainproof covers and temperature-control systems.

Among the several key influencing factors, water vapor can cause the PM_2.5_/PM_10_ sensor to overestimate the concentration of particulate matter [33,44], especially for hygroscopic particles, and further enhance the scattering effect of the particulate matter on light. Studies have reported that RH higher than 60%, 75%, or 85% can overestimate the referenced PM mass concentrations, varying by sensors’ properties, local climate, and particles’ delinquencies [45]. To make the validation more robust, we adjusted both RH and temperature in validation models for outdoor or indoor measurements. By comparison, after adjusting for RH, temperature, and measurement time lengths, the R^2^ of the outdoor models increased by 0.14 (from 0.71 to 0.85) and 0.23 (from 0.28 to 0.51) for PM_2.5_ and PM_10_, respectively, and the R^2^ of the indoor models increased by 0.10 (from 0.87 to 0.97) and 0.14 (from 0.78 to 0.92) for PM_2.5_ and PM_10_, respectively. All these increments were obtained in random forest (RF) regression models, and the increments were higher than those obtained by the multiple linear models (ML).

The ratio of PM_2.5_ to PM_10_ (k value) is an indicator suggesting whether PM_2.5_ accounted for a predominant proportion of PM_10_ [46,47,48]. A comparative study of various low-cost sensors found that low-cost aerosol sensors are unable to accurately differentiate particle sizes and can only provide linear measurements of aerosol concentration within the range of accumulation mode sizes [49]. However, different size fractions could cause interferences with the precise measurements. For example, the same PM_10_ concentration levels might be composed of different ratios of PM_2.5_ and PM_2.5–10_, particularly in outdoor air, with its mixture of particle emission sources. To mitigate the interference of particulate matters, especially in the complex outdoor environment with varying particle sizes, we added k value (the ratio of PM_2.5_/PM_10_) in our model. We observed in the outdoor validation of PM_10_ that the additional adjustment for k value in the RF model yielded an increase in R^2^ from 0.51 to 0.80 (Figure 3, Table S4).

Durability is another important factor in characterizing the sensor monitor since the abrasion of sensors was unavoidable over a long time period. In our study, we tested the sensor for a whole year continuously in an outdoor environment with more extreme climate conditions, and no maintenance or changes were performed for the sensor monitors during the one-year continuous measurement. The results showed that the sensor performance was almost not changed and not influenced in a year. This indicates that the instrument had a good durability and stability at least for one year of continuous measurement in the outdoor environment, which could be even longer in an indoor environment.

By comparing the validation by the optimal random forest model and the conventional linear regression model, we can see a large improvement either for PM_2.5_ or PM_10_ in the indoor and outdoor environment. Generally, after adjusting parameters, the random forest model had the best fit (R^2^ = 0.83), reaching a high level among those currently reported (R^2^ = 0.81−0.83) [38,40]. In detail, the internal consistency between sensors reached a high r ≥ 0.99 and the internal variability was lower than 2 μg/m^3^ (IDV ≤ 1.84 μg/m^3^) in measuring PM_2.5_ and PM_10_ in both the indoor and outdoor environment. The consistency between the sensors was significantly higher than in other studies (r ranged from 0.86 to 0.90) [38]. Compared to validation in the outdoor environment, the validation indicated the R^2^, MRE, and RMSE for both PM_2.5_ and PM_10_ measurements in the indoor environment were all better in the indoor environment. This was largely due to more mild and stable indoor measurements with less fluctuations of PM_2.5_, temperature, and RH. Both indoors and outdoors, the performance of PM_2.5_ (outdoor R^2^ = 0.9, indoor R^2^ = 0.97) was better than that of PM_10_ (outdoor R^2^ = 0.8, indoor R^2^ = 0.91). If applied in the estimation of human exposure to PM_2.5_ and PM_10_, the accuracy of the optimized PM_2.5_ and PM_10_ model misestimates are reduced by 2.28 μg/m^3^ and 8.72 μg/m^3^ in the outdoor measurement and 1.66 μg/m^3^ and 1.6 μg/m^3^ in the indoor measurement, respectively.

To the best of our knowledge, this study has as a strength that it integrated the application of sensor technology in simultaneous measurements of PM_2.5_ and PM_10_, and their long-term measurements were validated in multiple scenarios: both indoor and outdoor environments. An optimal validation method was identified with comprehensive consideration of climate factors, size distribution of fine and respirable particulate matter, and sensor durability.

There are some limitations in this study. First, we acknowledge that our sensor monitor might not completely control the influences of water vapor by adjusting RH in the model since the complexity of water vapor in influencing the sensor monitors involves the particle delinquencies, particle size distribution, particle composition, and instrument service time. This deserves more research to further reduce and even remove the influences of RH, e.g., by integrating a lightweight heating device or filter membrane to the sensor. Second, we obtained only hourly PM_2.5_ and PM_10_ with the TEOM instrument in the outdoor measurement as the reference data, which was not at the same minute-level time resolution as in the indoor environment against the TSI instrument. Third, PM_10_ was not compared with the indoor MiniVol, but it was compared with the outdoor TEOM, and both instruments (MiniVol and TEOM) were validated against the gravity method. Fourth, the indoor comparison was only conducted in the summer and did not cover the whole year, but we conducted a long-term comparison outdoors for one continuous year, which showed greater advantages for machine learning methods. Lastly, we noticed and put all sensors at the same distance surrounding the reference instrument, but it could still be occasionally disturbed by the uneven distribution of particles dominating in a certain direction. We also noticed that the performance of sensor measurements was different in different environmental scenes. Although we have included the typical scenarios in our studies, application in other environment is suggested for prior testing and validation to accompany the field tests.

## 5. Conclusions

Our study evaluated and optimized the performance of sensor monitors in measuring PM_2.5_ and PM_10_ under typical emission scenarios both indoors and outdoors. Sensor monitors (FS-Air 2.0) provided reliable measurements of PM_2.5_ and PM_10_ with high accuracy and acceptable inter and intra-device consistency under typical indoor and outdoor scenarios. The RF model performed best among the six machine learning models, adjusting for influencing factors including temperature, RH, particulate matter ratio (k value, PM_2.5_/PM_10_), and measurement time duration. Our study helps enhance the data quality of new, low-cost PM monitors; promote the exploration of its applicability in various indoor and outdoor scenarios in scientific research; and further facilitate an economical and accurate assessment of human exposure to particulate matter in environment and health studies.

## Figures and Tables

**Table 1:** Comparisons of parallel measurements on outdoor PM_2.5_ and PM_10_ (mean ± SD, range) by sensor monitors and TEOM for a whole year.

	Measurement Days (Sensors/TOEM)	PM_2.5_ (μg/m^3^)			PM_10_ (μg/m^3^)		
		Sensors	TEOM	R^2^	Sensors	TEOM	R^2^
All year	374/372	38.3 ± 25.6 *	33.3 ± 23.6	0.79	45.4 ± 29.5 *	54.7 ± 38.4	0.26
		(0.0~180.6)	(1~211.0)		(0.5~210.3)	(0.0~493.0)	
Spring (1 March–30 May)	92/91	43.4 ± 22.5 *	35.0 ± 20.5	0.69	52.3 ± 25.9 *	60.6 ± 45.7	0.04
		(3.5~172.8)	(1.0~142.0)		(4.3~191.6)	(0.0~493.0)	
Summer (1 June–31 August)	89/90	25.3 ± 15.7 *	21.1 ± 12.6	0.72	30.1 ± 19.4 *	38.8 ± 18.2	0.22
		(0.7~87.9)	(1.0~95.0)		(1.7~104.7)	(0.00~111.0)	
Autumn (1 September–30 November)	91/89	29.4 ± 20.2	29.0 ± 18.4	0.73	34.8 ± 23.6 *	56.3 ± 31.4	0.41
		(0.0~113.2)	(1.0~146.0)		(0.0~124.6)	(0.0~323.0)	
Winter (1 December–28 February)	102/101	52.8 ± 30.1 *	46.2 ± 30.1	0.80	62.1 ± 33.6	60.9 ± 37.8	0.42
		(2.5~180.6)	(2.0~211.0)		(3.3~210.3)	(0.0~363.0)	

Note: The average (±SD) temperature and RH in the whole year were 19.5 ± 8.5°C and 67.2 ± 14.6%. In the spring season, they were 17.9 ± 5.3 °C and 68.6 ± 15.3%. In summer, they were 28.7 ± 3.2°C and 74.9 ± 11.4%. In autumn, they were 22.9 ± 6.3 °C and 65.2 ± 13.8%, and in winter, they were 9.8 ± 3.7 °C and 61.3 ± 14.0%. * Compared with TEOM, *p* < 0.05. R^2^ is calculated using a linear model.

**Table 2:** Comparisons of parallel measurements on indoor PM_2.5_ and PM_10_ (mean ± SD, range) by sensors and TSI in offices and residences.

			PM_2.5_ (μg/m^3^)			PM_10_ (μg/m^3^)		
	Indoor Sources	Measurement Time	Sensors	TSI	R^2^	Sensors	TSI	R^2^
Office	No obvious emission sources	60 days	17.6 ± 13.3 *	19.9 ± 14.3	0.66	20.4 ± 16.0 *	27.1 ± 18.8	0.64
			(0.3~58.9)	(0.8~91.5)		(0.4~71.8)	(1.9~236.6)	
Residence	Mosquito-repellent	130 min	67.4 ± 55.9 *	83.5 ± 82.9	0.98	73.6 ± 56.2 *	107.5 ± 105.0	0.98
			(25.5~222.7)	(25.1~305.3)		(28.3~229.8)	(33.0~388.1)	
	Worship incense	110 min	34.1 ± 42.7 *	58.7 ± 76.9	0.98	37.3 ± 44.7 *	77.2 ± 98.6	0.97
			(2.7~139.2)	(8.5~254.7)		(3.8~145.0)	(11.2~328.2)	
	Candle	115 min	23.8 ± 12.0 *	27.5 ± 14.5	0.96	28.6 ± 16.0 *	38.5 ± 18.7	0.97
			(10.5~73.3)	(16.5~87.2)		(12.7~88.5)	(22.8~113.7)	
	Cooking	150 min	27.7 ± 28.5 *	47.1 ± 60.0	0.98	36.3 ± 40.0 *	66.1 ± 84.0	0.98
			(8.7~150.7)	(14.2~327.5)		(10.3~212.2)	(19.7~465.2)	
	Smoking	151 min	58.3 ± 63.0 *	62.4 ± 66.9	0.99	82.8 ± 84.6 *	65.0 ± 66.4	0.97
			(10.0~308.2)	(19.5~370.7)		(27.7~471.6)	(11.2~326.3)	

Note: The measurements were performed at an average room temperature of 31.2 ± 1.1 °C, with the average RH and atmospheric pressure of 69.2 ± 4.3% and 100.3 ± 0.5 Pa, respectively. * Compared with TSI, *p* < 0.05. R^2^ is calculated using a linear model.

**Table 3:** The regression R^2^ of PM_2.5_ and PM_10_ by sensor monitors against reference instruments in different machine learning models (10-fold cross-validation).

	References	Particles	NM	ML	SVM	MLP	DT	KNN	RF
Outdoor (hourly averages)	TEOM	PM_2.5_	0.79	0.82	0.85	0.84	0.84	0.83	0.90
	TEOM	PM_10_	0.26	0.33	0.23	0.41	0.59	0.76	0.80
Indoor (minutely averages)	TSI	PM_2.5_	0.66	0.84	0.85	0.90	0.95	0.93	0.97
	TSI	PM_10_	0.64	0.77	0.77	0.80	0.85	0.84	0.91

Note: A multi-variate regression in different machine learning models was applied, adjusting for temperature, RH, measurement time length (months), and k value. NM, normal model (single factor linear model); ML, multiple linear model; SVM, support vector machines model; MLP, multi-layer perceptron (neural network model); DT, decision tree model; KNN, K-nearest neighbor model; RF, random forest model.

## Data Availability

Data can be accessed under certain conditions. Interested researchers can request access through the authors and provide a detailed description of their research objectives and data usage plans.

## Supplementary Materials

The following supporting information can be downloaded at: https://www.mdpi.com/article/10.3390/s24113448/s1, Figure S1: The scatter plot of PM2.5 and PM10 by sensors monitors against reference instrument adjusted for covariates stepwise in ML and RF; Table S1: Parallel measurements and comparisons of hourly averages of PM10 by TSI and TEOM by linear regression model; Table S2: Parallel measurements and comparisons of daily averages of PM2.5 by TSI and MiniVol gravity method by linear regression model; Table S3: Comparisons of PM2.5 and PM10 between sensor monitors and TEOM in outdoor air in the whole year and in subgroup days with PM2.5 and PM10 as the primary pollutant by linear regression model, respectively; Table S4: The regression R2 of PM2.5 and PM10 by sensor monitors against reference data adjusted for covariates by ML and RF(10-fold cross-validation); Table S5: Indoor and outdoor raw and RF validated measurements of PM2.5 and PM10 by sensor monitors.
