# Supplementary material for: Multi-Scenario Validation and Assessment of a Particulate Matter Sensor Monitor Optimized by Machine Learning Methods
Source: Sensors (Basel). 2024 May 27;24(11):3448. doi: 10.3390/s24113448 (PMC11174656; doi:10.3390/s24113448)
Supplement: Supplementary file 1 [file sensors-24-03448-s001.zip › sensors-2990279-supplementary.pdf]

Supplementary materials

Multi-scenario validation and assessment of a particulate matter sensor monitor optimized by machine learning methods

Table S1 Parallel measurements and comparisons of hourly averages of PM_{10} by TSI and TEOM by linear regression model

Table S2. Parallel measurements and comparisons of daily averages of $PM_{2.5}$ by TSI and MiniVol gravity method by linear regression model

Table S3. Comparisons of $PM_{2.5}$ and PM_{10} between sensor monitors and TEOM in outdoor air in the whole year and in subgroup days with $PM_{2.5}$ and PM_{10} as the primary pollutant by linear regression model, respectively.

Table S4. The regression R^2 of $PM_{2.5}$ and PM_{10} by sensor monitors against reference data adjusted for covariates by ML and RF(10-fold cross-validation)

Table S5. Indoor and outdoor raw and RF validated measurements of $PM_{2.5}$ and PM_{10} by sensor monitors

Figure S1. The scatter plot of $PM_{2.5}$ and PM_{10} by sensors monitors against reference instrument adjusted for covariates stepwise in ML and RF.

Table S1 Parallel measurements and comparisons of hourly averages of PM_{10} between TSI and TEOM by linear regression model

	Measurement hours	Mean ± SD	Range	R^2	RMSE
TEOM	1201.0	67.3±36.8	11.0~274.0	0.91	5.7
TSI	1201.0	69.9±38.7	20.4~262.5		
Temperature	1201.0	13.2±5.2	1.3~28.0		
Relative humidity	1201.0	49.8±21.4	5.8~94.1		

Note: RMSE: Root Mean Square Error; Equation between TSI and TEOM: y(TEOM)=0.49x(TSI)+2.22

Table S2 Parallel measurements and comparisons of daily averages of $PM_{2.5}$ between TSI and MiniVol gravity method by linear regression model.

	Measurement days	Mean ± SD	Range	R^2	RMSE
MiniVol TAS	49.0	19.3±9.1	1.9~41.6	0.90	4.9
TSI	49.0	20.9±12.0	7.2~51.3		
Temperature	49.0	31.2±1.1	28.6~32.9		
Relative humidity	49.0	69.2±4.3	60.8~81.2		

Note: RMSE: Root Mean Square Error; Equation between TSI and MiniVol: y(MiniVol)=0.996x(TSI)-1.01

Table S3. Comparisons of $PM_{2.5}$ and PM_{10} between sensor monitors and TEOM in outdoor air in the whole year and in subgroup days with $PM_{2.5}$ and PM_{10} as the primary pollutant by linear regression model, respectively.

	**Measurement days**	**$PM_{2.5}$**			**PM_{10}**		
		R^2	RMSE	MRE	R^2	RMSE	MRE
**All year**	365.0	0.79	12.80	40.02	0.33	33.90	50.72
**$PM_{2.5}$ as the primary pollutant**	157.0	0.74	15.62	33.75	0.30	39.72	62.78
**PM_{10} as the primary pollutant**	208.0	0.65	10.19	44.71	0.36	28.83	43.42

Note: RMSE: Root Mean Square Error; MRE: Mean Relative Error; $PM_{2.5}$ as the primary pollutant: the Individual Air Quality Index (IAQI) of $PM_{2.5}$ is greater than that of PM_{10}; PM_{10} as the primary pollutant: the Individual Air Quality Index (IAQI) of PM_{10} is greater than that of $PM_{2.5}$. IAQI is calculated according to the Ambient Air Quality Standard in China (GB 3095-2012) (The formula for calculating the Indoor Air Quality Index (IAQI) is as follows: IAQI = (Cp - Bp) / (Tp - Bp) * (Hi - Lo) + Lo, Where: IAQI represents the Indoor Air Quality Index. Cp is the measured concentration of a specific pollutant. Bp is the lower bound of the concentration range for that pollutant. Tp is the upper bound of the concentration range for that pollutant. Hi is the upper bound of the IAQI scale for that pollutant. Lo is the lower bound of the IAQI scale for that pollutant. By applying this formula to each pollutant category, we can determine their respective IAQI values.).

Table S4. The regression R^2 of $PM_{2.5}$ and PM_{10} by sensor monitors against reference data adjusted for covariates by ML and RF(10-fold cross-validation)

		ML regression			RF regression		
		Model 1	Model 2	Model 3	Model 1	Model 2	Model 3
**Outdoor(hour)**	$PM_{2.5}$	0.77	0.80	0.82	0.71	0.85	0.90
	PM_{10}	0.11	0.29	0.33	0.28	0.51	0.80
**Indoor(min)**	$PM_{2.5}$	0.82	0.82	0.84	0.87	0.97	0.97
	PM_{10}	0.76	0.76	0.76	0.78	0.92	0.91

Note： Model 1: yPM_{ref}~XPM_{sensor}; Model 2: yPM_{ref}~XPM_{sensor}+T+RH+month; Model 3: yPM_{ref}~XPM_{sensor}+T+RH+month+k

ML: Multiple regression model; RF: Random forest regression model

Table S5. Indoor and outdoor raw and RF validated measurements of $PM_{2.5}$ and PM_{10} by sensor monitors

		Intra-device comparisons within sensor monitors				Inter-device comparisons between sensor monitors and reference instruments					
	**Particles**	**Intra-device Correlation coefficient (r)**		**Intra-device Variability (IDV, μg/m³)**		**Coefficient of determination (R^2)**		**Mean relative error (MRE/%)**		**Root Mean Square Error (RMSE, μg/m³)**	
		CL	RF	CL	RF	CL	RF	CL	RF	CL	RF
**Outdoor**	$PM_{2.5}$	0.99	0.99	1.1	1.4	0.78	0.90	40.02	18.60	12.8	5.6
**(Hourly averages)**	PM_{10}	0.99	0.99	1.1	1.4	0.33	0.80	50.72	24.33	33.9	17.5
**Indoor**	$PM_{2.5}$	0.98	0.99	2.0	1.8	0.69	0.97	37.28	8.80	9.4	1.9
**(Minutely averages)**	PM_{10}	0.97	0.99	3.0	1.7	0.66	0.91	40.54	13.54	14.0	4.6

CL: Validated by conventional linear regression models for all data; RF: random forest model(10-fold cross-validation)

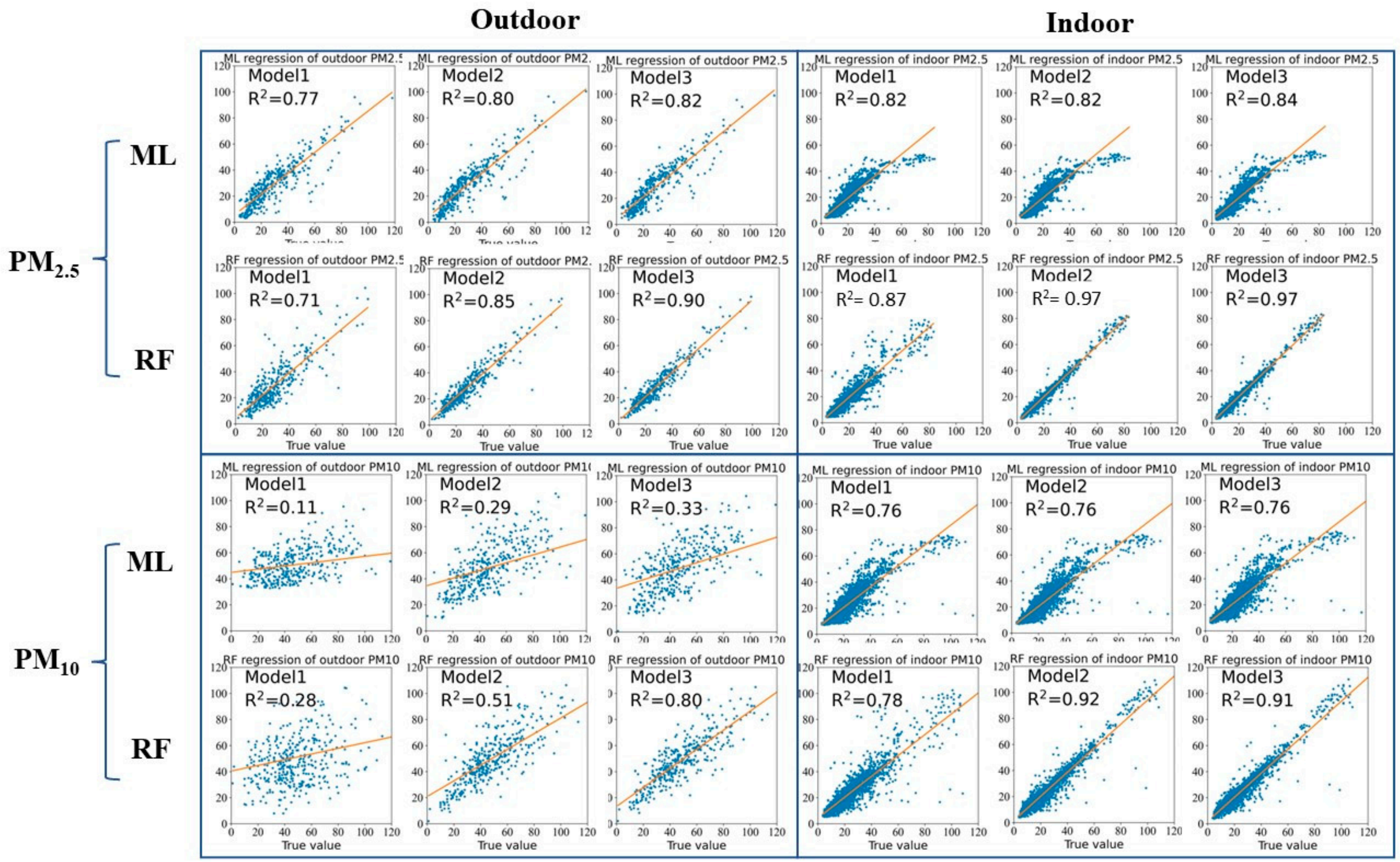

Outdoor
Indoor
PM2.5
PM10
ML
RF
ML regression of outdoor PM2.5
ML regression of indoor PM2.5
RF regression of outdoor PM2.5
RF regression of indoor PM2.5
ML regression of outdoor PM10
ML regression of indoor PM10
RF regression of outdoor PM10
RF regression of indoor PM10
Model1
Model2
Model3
R2=0.77
R2=0.80
R2=0.82
R2=0.82
R2=0.82
R2=0.84
R2=0.71
R2=0.85
R2=0.90
R2= 0.87
R2= 0.97
R2=0.97
R2=0.11
R2=0.29
R2=0.33
R2=0.76
R2=0.76
R2=0.76
R2=0.28
R2=0.51
R2=0.80
R2=0.78
R2=0.92
R2=0.91
True value

Figure S1 The scatter plot of $PM_{2.5}$ and PM_{10} by sensors monitors against reference instrument adjusted for covariates stepwise in ML and RF.

ML: Multiple regression model; RF: Random forest regression model; Model 1: yPM_{ref}~XPM_{sensor}; Model 2: yPM_{ref}~XPM_{sensor}+T+RH+month; Model 3: yPM_{ref}~XPM_{sensor}+T+RH+month+k
